# Preparation and Characterization of Neutrophil Membrane-Fused Mitochondria (nMITO)

**DOI:** 10.21769/BioProtoc.5744

**Published:** 2026-07-20

**Authors:** Qing Zhang, Yuqin Yue, Xing Zhou

**Affiliations:** Yunnan Key Laboratory of Stem Cell and Regenerative Medicine, Rehabilitation School, Kunming Medical University, Kunming, China

**Keywords:** nMITO, Mitochondria isolation, Neutrophil membranes, Membrane coating, Mitochondrial assessment

## Abstract

Mitochondrial transplantation is an emerging strategy for cellular repair, yet its efficiency is often limited by poor targeting and environmental instability. This protocol details the fabrication and comprehensive characterization of neutrophil membrane-fused mitochondria (nMITO), a hybrid organelle platform designed to combine the metabolic vigor of natural mitochondria with the targeting and anti-inflammatory properties of neutrophil membranes. We describe an optimized workflow for mouse heart mitochondrial isolation, lipopolysaccharide (LPS)-activated neutrophil membrane (NEM) extraction, and the subsequent sonication-mediated fusion process. Characterization techniques include dynamic light scattering (DLS) for size and zeta potential, transmission electron microscopy (TEM) for ultrastructural integrity, and bioenergetic assays [ATP synthesis and tetramethylrhodamine methyl ester (TMRM)-based membrane potential] to ensure functional preservation.

Key features

• The protocol provides a methodology for the isolation of neutrophil membranes from mouse bone marrow and mitochondria from the heart.

• The protocol provides a methodology for the fabrication of neutrophil membrane-fused mitochondria (nMITO).

## Graphical overview


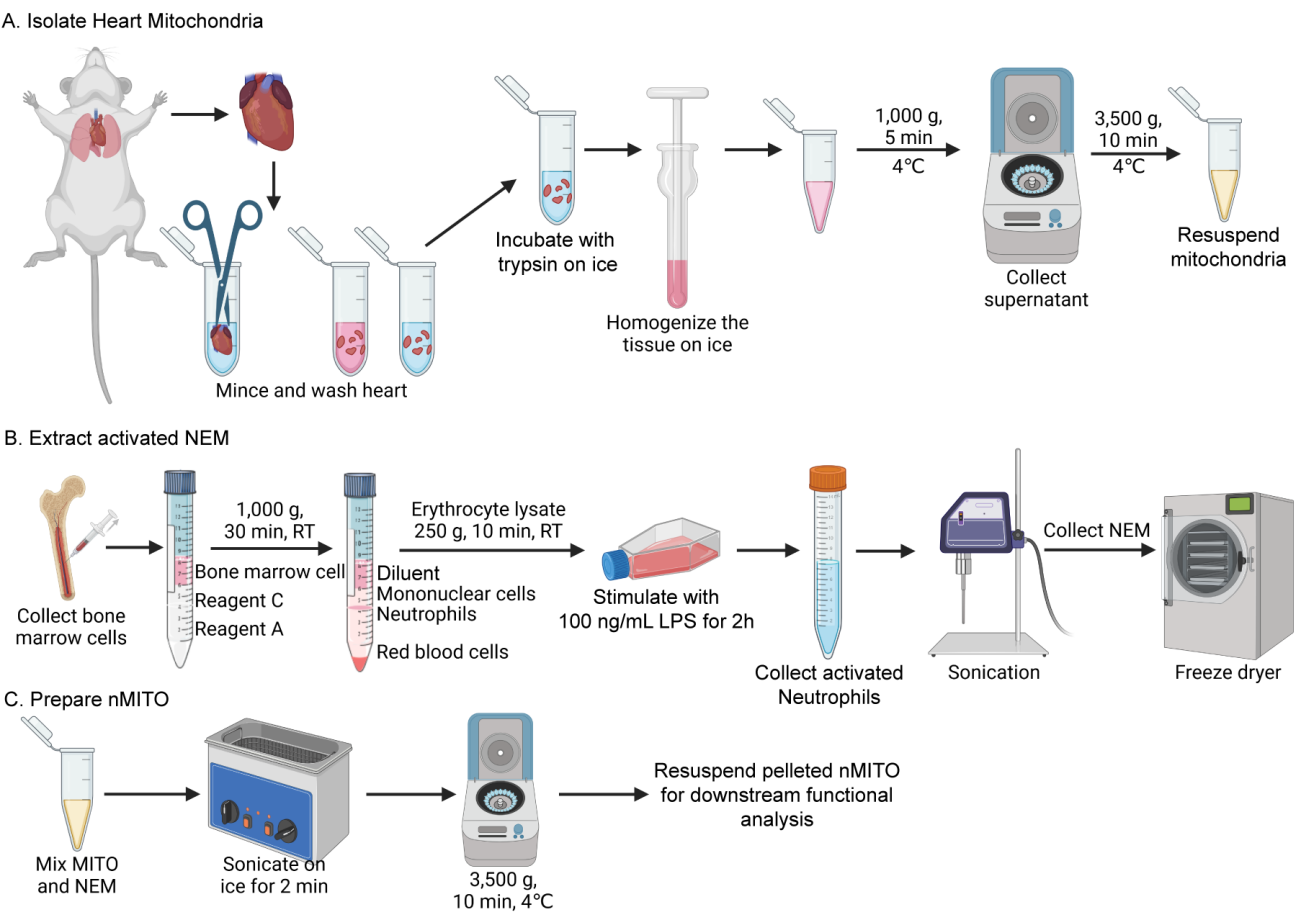


**Schematic of the preparation of neutrophil membrane-fused mitochondria (nMITO).** Isolation of heart mitochondria and extraction of activated neutrophil membranes from the bone marrow, followed by ice-bath sonication-mediated fusion. Created in BioRender.

## Background

Mitochondrial transplantation has emerged as a promising method to restore cellular homeostasis [1,2]; however, “naked” mitochondria lack the ability to target specific inflammatory sites [3,4]. Neutrophils are naturally home to sites of injury via β-integrin/ICAM-1 interactions and carry receptors capable of neutralizing inflammatory cytokines [5,6]. By fusing neutrophil membranes onto isolated mitochondria, neutrophil membrane-fused mitochondria (nMITO) integrates the internal bioenergetic machinery of the mitochondria with the surface-level biological functionality of the neutrophil. In this hybrid system, the mitochondria provide ATP, while the neutrophil membrane facilitates targeted “homing” and “protective” functions. To maximize these capabilities, lipopolysaccharides (LPSs) are utilized in this protocol to activate neutrophils prior to membrane extraction. This activation significantly upregulates the expression of chemokine receptors, inflammatory cytokine receptors, and β-integrins. Consequently, the resulting neutrophil membrane (NEM) can effectively adsorb and neutralize pro-inflammatory cytokines while utilizing the enhanced β-integrin/ICAM-1 interaction for precise site-specific homing.

The preparation of nMITO consists of three primary stages: (i) isolation of mitochondria from mouse heart tissue via differential centrifugation; (ii) extraction of activated neutrophil membranes (NEM) from LPS-stimulated bone marrow neutrophils; (iii) fabrication of nMITO through ice-bath sonication-mediated fusion of the two components. Following the preparation, basic characterization is performed to evaluate the physicochemical properties, bioenergetic functions, and protein marker inheritance of the resulting nMITO.

## Materials and reagents

**Biological materials**

1. Male C57BL/6J mice (6–8 weeks old) for the isolation of heart mitochondria and bone marrow neutrophils

**Reagents**

1. LPS (Sigma, catalog number: L2630)

2. Tissue Mitochondrial Isolation kit (Beyotime, catalog number: C3606)

3. Mouse Bone Marrow Neutrophil Isolation kit (Solarbio, catalog number: P8550)

4. ATP Assay kit (Beyotime, catalog number: S0027)

5. TMRM (MCE, catalog number: HY-D0984A)

6. CCCP (MCE, catalog number: HY-100941)

7. PBS (Beyotime, catalog number: C0221A)

8. Red blood cell lysis buffer (Beyotime, catalog number: C3702)

9. EDTA (Beyotime, catalog number: ST1305)

10. Mannitol (CHRON CHEMICALS, CAS number: 69-65-8)

11. Sucrose (MCE, catalog number: HY-B1779)

12. 1 M Tris-HCl, PH 7.4 (Beyotime, catalog number: ST786)

13. Protease inhibitor (Beyotime, catalog number: P1005)

14. RPMI 1640 medium (Gibco, catalog number: 11875093)

15. Bradford Protein Assay kit (Beyotime, catalog number: P0006)

16. COX IV monoclonal antibody (Proteintech, catalog number: 66110-1-Ig)

17. Beta actin monoclonal antibody (Proteintech, catalog number: 66009-1-Ig)

18. IL-6R polyclonal antibody (Thermo Fisher Scientific, catalog number: PA5-102425)

19. CXCR2 antibody (Affinity, catalog number: DF7095)

**Solutions**

1. Neutrophil membrane (NEM) isolation buffer (see Recipes)

**Recipes**

**1. NEM isolation buffer**

**Table:** 

Reagent	Final concentration	Quantity or volume
Mannitol	225 mM	2.0494 g
Sucrose	75 mM	1.2835 g
Tris-HCl	30 mM	1.5 mL
EDTA	0.5 mM	7.306 mg
Protease inhibitor	1% (v/v)	0.5 mL

Prepare 50 mL of neutrophil membrane isolation buffer according to the recipe. Adjust the final volume to 50 mL with ddH_2_O and store at 4 °C.

**Laboratory supplies**

1. Centrifuge tubes (1.5 mL and 15 mL) (Servicebio, catalog numbers: EP-150X-J, EP-1501-J)

2. Culture flask (LABSELECT, catalog number: 13112A)

3. Cell scraper (Beyotime, catalog number: FSCP029)

4. 10 cm scissors and tweezers (Beyotime, catalog numbers: FS001, FS225)

5. 26 G disposable sterile syringe (Beyotime, catalog number: FS801)

## Equipment

1. Dounce homogenizer and pestles (glass, 2 mL) (Beyotime, catalog number: FGH002)

2. Centrifuge (angle and swing-out rotor) (CENCE, model: HT165R; HERMLE, model: Z32 HK)

3. Bath sonicator (SCIENTZ, model: SB25-12D)

4. Probe sonicator (LICHEN, model: LC-UP-400)

5. Cell culture incubator (PHCBI, model: MCO-170AICUVDL-PC)

6. Dynamic light scattering analyzer (Brookhaven, model: NanoBrook 90PlusPALS)

7. Flow cytometer (Agilent, model: NovoCyte Advanteon)

8. Vertical electrophoresis system (Bio-Rad, model: PowerPac Basic and mini PROTEAN^®^ Tetra cell)

9. Automatic Cell Analyzer (Countstar, model: Mira BF-S)

10. Freeze dryer (SCIENTZ, model: 10YG/B)

11. Multimode microplate reader (Agilent, model: Synergy H1)

12. Chemiluminescence imaging system (GE, model: Amersham Imager600)

## Software and datasets

1. NovoExpress (Agilent Technologies); used for flow cytometry data acquisition and analysis

2. Gen5 Microplate Reader and Imager Software (Agilent Technologies); used for ATP luciferase assay data acquisition and analysis

3. GraphPad Prism 10 (GraphPad Software); used for statistical analysis and data visualization

4. Particle Solutions (Brookhaven Instruments); used for dynamic light scattering (DLS) data acquisition and particle size/zeta potential analysis

5. ImageQuant TL (GE Healthcare); used for chemiluminescence image acquisition of western blots

6. Countstar Software (Countstar); used for automatic cell counting

## Procedure

**A. Isolation of heart mitochondria (MITO)**

1. Sacrifice the mouse by cervical dislocation, remove the heart, and immediately place it in an ice-cold tube containing PBS.

2. Rinse the heart 2–3 times with 4 mL of pre-cooled PBS until the blood is thoroughly removed.

3. Transfer the heart to a 1.5 mL centrifuge tube and record the tissue weight. Mince the heart into small pieces (approximately 1 mm^3^) using scissors. Add 10 volumes (v/w) of pre-cooled PBS and incubate on ice for 3 min. Centrifuge at 600× *g* for 20 s to pellet the tissue and discard the supernatant.

*Note: All centrifugation steps for mitochondria are performed at 4 °C using a fixed-angle rotor.*

4. Add 8 volumes of trypsin solution (provided in the Tissue Mitochondrial Isolation kit) and incubate on ice for 20 min. Centrifuge at 600× *g* for 20 s and discard the supernatant.

5. Add 2 volumes of mitochondrial isolation reagent A (provided in the Tissue Mitochondrial Isolation kit) to resuspend the tissue to wash away residual trypsin. Centrifuge at 600× *g* for 20 s and discard the supernatant.

6. Add 8 volumes of pre-cooled mitochondrial isolation reagent A. Homogenize the tissue on ice using a Dounce homogenizer (10–15 strokes).

7. Centrifuge at 1,000× *g* for 5 min and then transfer the supernatant to a new centrifuge tube.

8. Centrifuge the supernatant at 3,500× *g* for 10 min at 4 °C. Carefully discard the supernatant to obtain the isolated mitochondria (MITO) pellet.

9. Resuspend the pellet in pre-cooled PBS (100–200 μL) and measure the mitochondrial protein concentration using the Bradford or BCA method.

**B. Extraction of activated NEM**

1. Detach the femurs and tibias and place them into ice-cold serum-free RPMI 1640 medium to maintain cell viability. Flush the medullary cavity with RPMI 1640 medium equilibrated to room temperature (RT) using a 26 G sterile disposable syringe until the bone segments appear white to ensure maximum marrow recovery.

2. Perform neutrophil purification according to the manufacturer’s instructions. In brief, follow a density gradient ratio of 4 mL of solution A to 2 mL of solution C for marrow samples less than 4 mL (solution A and C are provided in the Mouse Bone Marrow Neutrophil Isolation kit). Add Solution A to the bottom of the 15 mL centrifuge tube first. Tilt the tube and gently layer solution C on top of solution A, ensuring the interface remains undisturbed. Carefully add the marrow suspension [adjusted to 0.5–1 × 10^8^ cells/mL, using an automatic cell analyzer (Countstar Mira BF-S)] onto the surface of Solution C. Maintain the integrity of the layers throughout the process to ensure maximum separation efficiency. Centrifuge at 500–1,000× *g* for 20–30 min at RT using a swing-out rotor.

3. After centrifugation, collect the granulocyte layer along with approximately 0.5 mL of the isolation medium above and below it, and transfer the mixture to a new centrifuge tube. Add PBS to a final volume of 10 mL and centrifuge at 400× *g* for 10 min at RT (swing-out rotor).

4. (Optional) If red blood cell (RBC) contamination is visible in the pellet, add an appropriate volume of RBC lysis buffer and incubate for 5 min. Following lysis, centrifuge at 250× *g* for 5 min at RT (swing-out rotor).

5. Discard the supernatant, add 10 mL of PBS to resuspend the cells, and centrifuge at 250× *g* for 10 min at RT (swing-out rotor).

6. Discard the supernatant again and resuspend the final cell pellet in serum-free RPMI 1640 medium.

7. Incubate the purified neutrophils at a concentration of 1–2 × 10^7^ cells/mL in serum-free RPMI 1640 medium containing 100 ng/mL LPS for 2 h in the cell culture incubator (37 °C, 5% CO_2_).

8. Centrifuge the LPS-stimulated cells at 250× *g* for 10 min at RT (swing-out rotor). Resuspend the activated neutrophils in pre-cooled NEM isolation buffer at a concentration of approximately 5 × 10^7^ cells/mL. Perform probe sonication (100 W) for 5 min (2 s on/3 s off) on ice.

9. Centrifuge the homogenate at 10,000× *g* for 10 min at 4 °C (angle rotor) and collect the supernatant containing membrane fragments. Then, freeze-dry the sample and store at -80 °C for further use.

**C. Preparation of nMITO**

1. Resuspend freshly isolated MITO and NEM individually in pre-cooled PBS to a final protein concentration of 2 mg/mL for each component. Combine the two components at a 1:1 protein mass ratio within a 1.5 mL centrifuge tube.

2. Sonicate the mixture in an ice-water bath for 2 min (at 40 KHz).

3. Centrifuge the mixture at 3,500× *g* for 10 min at 4 °C. Discard the supernatant containing unfused NEM. Resuspend the nMITO pellet in pre-cooled PBS for downstream functional analysis.

## Data analysis

Data were analyzed using GraphPad Prism 10.3.1 and presented as mean ± SD (n ≥ 3). For multi-group comparisons, one-way ANOVA (homogeneity of variance) was applied. Statistical significance was defined as P < 0.05.

## Validation of protocol

**Characterization and functional validation of nMITO**

**Figure 1.**
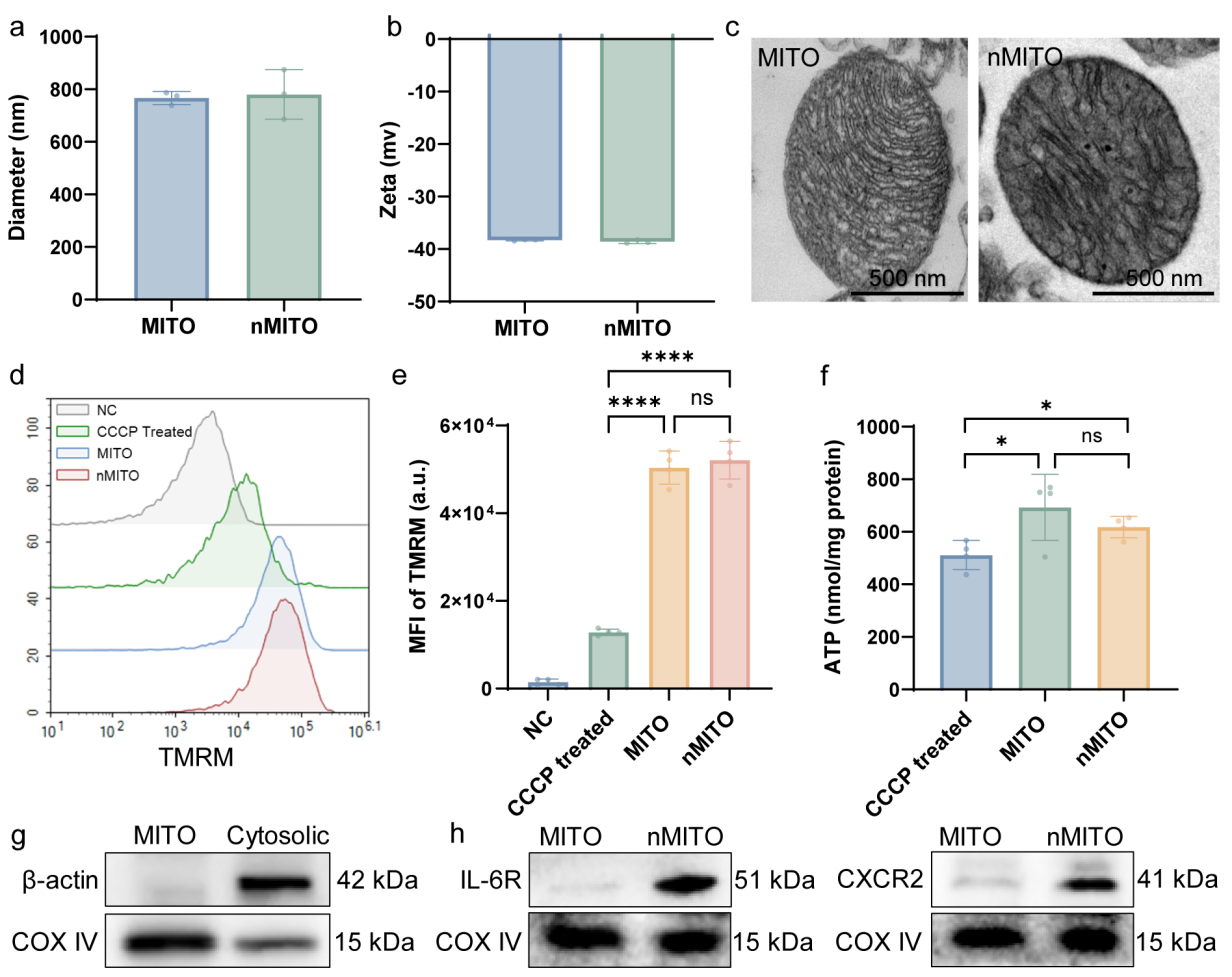
Physicochemical and functional characterization of neutrophil membrane-fused mitochondria (nMITO). **(a–b)** Physicochemical properties of mitochondria (MITO) and nMITO measured by dynamic light scattering (DLS), including (**a**) average hydrodynamic diameter and (**b**) surface zeta potential. (**c**) Representative transmission electron microscopy (TEM) images of MITO and nMITO. Scale bars = 500 nm. (**d–e**) Assessment of mitochondrial membrane potential (MMP) using TMRM staining. (**d**) Representative flow cytometry histograms showing the fluorescence distribution of TMRM. (**e**) Quantitative analysis of mean fluorescence intensity (MFI). NC: Unstained negative control; CCCP treated: MITO treated with 10 μM CCCP for 20 min as a depolarized control. (**f**) Quantification of ATP synthesis capacity in different groups. Data are presented as mean ± SD. Statistical significance was determined by one-way ANOVA. * and **** denote P < 0.05 and P < 0.0001, respectively. (**g**) Western blot analysis of isolation purity. COX IV (mitochondrial marker) and β-actin (cytoskeletal/cytosolic marker) were used to evaluate the separation efficiency between mitochondrial and cytosolic fractions. (**h**) Validation of neutrophil membrane inheritance on nMITO via western blot analysis. The presence of IL-6R and CXCR2 (activated neutrophil membrane markers) confirms successful fusion.

1. The particle size and zeta potential of nMITO were characterized using NanoBrook 90Plus PALS dynamic light scattering analyzer (DLS), while their morphology was visualized via transmission electron microscopy (TEM) (Figure 1a–c). Our results indicate that the fusion of neutrophil membranes does not significantly alter the particle size, surface charge, or morphological characteristics of the mitochondria, demonstrating that the structural integrity of the mitochondria was well-preserved throughout the fabrication process.

2. To evaluate mitochondrial functionality, the mitochondrial membrane potential (MMP) and ATP levels of MITO and nMITO were quantified, using CCCP-treated MITO (10 μM, 20 min) as a depolarized negative control. MMP was analyzed by measuring TMRM (100 nM, 30 min) fluorescence via flow cytometry (NovoCyte Advanteon, Agilent Technologies). MITO and nMITO exhibited high mean fluorescence intensity (MFI), which was significantly greater than that of the CCCP-treated group (Figure 1d–e). Furthermore, ATP synthesis was measured via a luciferase assay using a Synergy H1 multimode microplate reader (Agilent Technologies), where nMITO maintained high production rates comparable to native MITO (Figure 1f). These findings demonstrate that the bioenergetic functions of the mitochondria are not compromised by the membrane fusion.

3. Mitochondrial purity was assessed by western blot analysis, evaluating the expression of marker proteins in both mitochondrial and cytosolic fractions. COX IV served as the mitochondrial marker, while β-actin was used as the cytoskeletal/cytosolic marker to verify separation efficiency. Additionally, IL-6R and CXCR2 were used as markers for the activated neutrophil membrane (Figure 1g–h).

This protocol has been used and validated in the following research article:

- Qing, Z. et al. [7] Immunoengineered mitochondria for efficient therapy of acute organ injuries via modulation of inflammation and cell repair. *Science advances.*

## General notes and troubleshooting

**Troubleshooting**

**Problem 1:** Low yield or poor bioactivity of isolated mitochondria.

Possible causes: Excessive homogenization (causing mechanical damage) or elevated temperatures during the isolation process.

Solutions: Reduce the number of homogenization strokes or the duration of grinding. Ensure that all procedures are performed strictly on ice and that all buffers and equipment are pre-cooled to 4 °C to maintain mitochondrial structural and functional integrity.

**Problem 2:** Low purity of isolated neutrophils.

Possible causes: Reagents were not equilibrated to room temperature; use of standard (high-binding) centrifuge tubes; inappropriate centrifuge acceleration and deceleration settings.

Solutions: Ensure all separation reagents reach room temperature before use. Use low-protein-binding centrifuge tubes and a swing-bucket rotor to improve separation efficiency. Carefully layer the reagents (e.g., AC liquid) to ensure distinct interfaces. To minimize layer disturbance, set the centrifuge acceleration rate to 1 and the deceleration rate (brake) to 0.
